# *trans*-Cyclo­hexane-1,4-diyl bis­(4-nitro­phen­yl) dicarbonate

**DOI:** 10.1107/S1600536807066007

**Published:** 2007-12-18

**Authors:** Syed Nawazish Ali, Sabira Begum, Mitchell A. Winnik, Alan J. Lough

**Affiliations:** a H. E. J. Research Institute of Chemistry, International Center for Chemical Sciences, University of Karachi, Karachi 75270, Pakistan; b Department of Chemistry, University of Toronto, Toronto, Ontario, M5S 3H6, Canada

## Abstract

In the title crystal structure, C_20_H_18_N_2_O_10_, there are two independent mol­ecules, both of which lie on crystallographic inversion centres. In one mol­ecule the 4-nitro­phenyl dicarbonate groups are substituted in equatorial (*A*_eq_) positions of the chair-form cyclo­hexane ring while in the other mol­ecule the substitution is axial (*B*_ax_). The dihedral angles between the atoms of the symmetry-unique carbonate group (O=CO_2_—) and benzene ring for each mol­ecule are 47.3 (1)° for *A*_eq_ and 11.7 (2)° for *B*_ax_. In *B*_ax_, this facilitates the formation of a weak intra­molecular C—H⋯O hydrogen bond, while the packing is stabilized by weak inter­molecular C—H⋯O inter­actions.

## Related literature

For related literature, see: Ali *et al.* (2008 ▶).
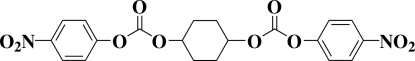


## Experimental

### 

#### Crystal data

C_20_H_18_N_2_O_10_
*M* *_r_* = 446.36
Triclinic, 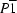

*a* = 7.6804 (14) Å
*b* = 11.6548 (18) Å
*c* = 12.3092 (11) Å
α = 63.201 (8)°
β = 87.254 (10)°
γ = 82.310 (7)°
*V* = 974.6 (3) Å^3^
*Z* = 2
Mo *K*α radiation
μ = 0.12 mm^−1^
*T* = 150 (1) K
0.22 × 0.20 × 0.08 mm

#### Data collection

Nonius KappaCCD diffractometer
Absorption correction: multi-scan (*SORTAV*; Blessing, 1995 ▶) *T*_min_ = 0.768, *T*_max_ = 0.996
7088 measured reflections
3355 independent reflections
1633 reflections with *I* > 2σ(*I*)
*R*_int_ = 0.080

#### Refinement

*R*[*F* ^2^ > 2σ(*F* ^2^)] = 0.063
*wR*(*F* ^2^) = 0.190
*S* = 0.96
3355 reflections
289 parameters
H-atom parameters constrained
Δρ_max_ = 0.30 e Å^−3^
Δρ_min_ = −0.27 e Å^−3^

### 

Data collection: *COLLECT* (Nonius, 2002 ▶); cell refinement: *DENZO-SMN* (Otwinowski & Minor, 1997 ▶); data reduction: *DENZO-SMN*; program(s) used to solve structure: *SIR92* (Altomare *et al.*, 1994 ▶); program(s) used to refine structure: *SHELXTL/PC* (Sheldrick, 2001 ▶); molecular graphics: *PLATON* (Spek, 2003 ▶); software used to prepare material for publication: *SHELXTL/PC*.

## Supplementary Material

Crystal structure: contains datablocks global, I. DOI: 10.1107/S1600536807066007/hb2675sup1.cif

Structure factors: contains datablocks I. DOI: 10.1107/S1600536807066007/hb2675Isup2.hkl

Additional supplementary materials:  crystallographic information; 3D view; checkCIF report

## Figures and Tables

**Table 1:** Hydrogen-bond geometry (Å, °)

*D*—H⋯*A*	*D*—H	H⋯*A*	*D*⋯*A*	*D*—H⋯*A*
C10*A*—H10*A*⋯O5*A*^i^	0.95	2.32	3.205 (6)	155
C6*B*—H6*BA*⋯O2*B*	0.95	2.24	2.812 (5)	118
C9*B*—H9*BA*⋯O2*A*^ii^	0.95	2.48	3.184 (5)	131

Symmetry codes: (i) 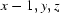; (ii) 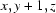.

**Fig. 1.**
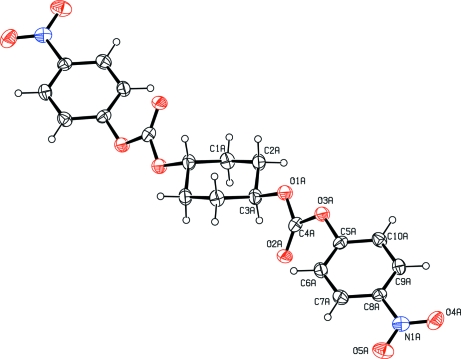
View of one of the independent molecules of the title compound with displacement ellipsoids drawn at the 30% probability level. Unlabeled atoms are related by the symmetry operator (-x, -y, 1 - z).

**Fig. 2.**
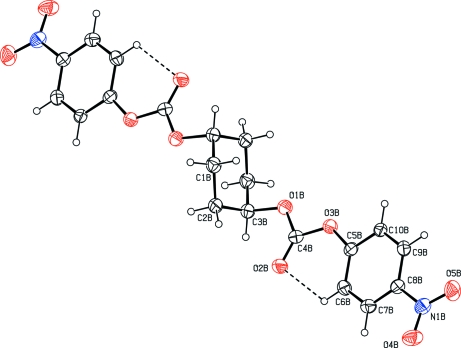
View of the other independent molecule of the title compound with displacement ellipsoids drawn at the 30% probability level. Unlabeled atoms are related by the symmetry operator (2 - x, 1 - y, -z). The dashed line indicates a hydrogen bond.

## supplementary crystallographic information

### Comment

The synthesis of title compound is similar to that of cyclohex-2-ene-1,4-diylbis(4-nitrophenyl)dicarbonate (Ali *et al.*, 2008). Here, we used a mixture of *cis* and *trans* isomers of cyclohexane-1,4-diol. The *trans* isomer has been separated from the mixture of *cis* and *trans* isomers. Most of the *trans* isomer remained undissolved in EtOH during the recrystallization at 358 K, after 40 minutes. Pale yellow plates of (I) were obtained after solubilizing this EtOH insoluble solid in dichloromethane. The molecular structure is illustrated in Figs. 1 and 2, showing that one of the two asymmetric molecules possesses equatorial substituents and the other axial. Within the latter, a weak C—H···O interaction (Table 1) occurs. Further C—H···O links help to establish the packing.

### Experimental

A solution of 4-nitrophenylchloroformate (5.64 g, 28.0 mmol) in dry dichloromethane (40 ml) was added dropwise *via* a 100 ml separating funnel into a solution of cyclohexane-1,4-diol (*cis* and *trans* isomers) (1.63 g, 14.0 mmol) in anhydrous pyridine (2.15 g, 2.2 ml, 27.1 mmol) and dry dichloromethane (20 ml) in a 250 ml round-bottom flask. A white suspension appeared which was allowed to stir gently at room temperature for 16 h. After this time more dry dichloromethane (40 ml) was added, which dissolved the suspension and then the reaction mixture was stirred for another 6 h. Then it was quenched by adding deionized water (40 ml). The reaction mixture was transferred to a separating funnel (500 ml), and the lower organic phase was removed. The aqueous phase was washed with dichloromethane (30 ml × 2), and the dichloromethane solutions were combined. These were then washed with deionized water (30 ml × 2), a 1.0% solution of acetic acid (50 ml × 2) and once more with deionized water (40 ml × 2), and then dried over anhydrous magnesium sulfate and filtered. After filtration, the solvent was removed by rotary evaporator. The product was dried in air overnight in a fume hood and then in a vacuum oven for 24 h at room temperature (< 1 Torr). The desired product was obtained in good yield (6.2 g, 84.0%) as a white solid. For recrystallization, the solid was dissolved in 95% EtOH (50 ml) at 358 K, after 40 minutes some of the solid (about 40%) remained undissolved. The warm solution was filtered and the EtOH-insoluble solid was recovered from the filter paper and dissolved in dichloromethane. Pale yellow plates of (I) were obtained by slow evaporation of solvent at room temperature.

### Refinement

The H atoms were placed in calculated positions, with C—H = 0.95–1.00 Å, and refined as riding, with *U*_iso_(H) = 1.2*U*_eq_(C).

### Crystal data

**Table:**

C_20_H_18_N_2_O_10_	*Z* = 2
*M**_r_* = 446.36	*F*_000_ = 464
Triclinic, *P*1	*D*_x_ = 1.521 Mg m^−^^3^
Hall symbol: -P 1	Mo *K*α radiation λ = 0.71073 Å
*a* = 7.6804 (14) Å	Cell parameters from 7088 reflections
*b* = 11.6548 (18) Å	θ = 2.7–25.2º
*c* = 12.3092 (11) Å	µ = 0.12 mm^−^^1^
α = 63.201 (8)º	*T* = 150 (1) K
β = 87.254 (10)º	Plate, pale yellow
γ = 82.310 (7)º	0.22 × 0.20 × 0.08 mm
*V* = 974.6 (3) Å^3^	

### Data collection

**Table:**

Nonius KappaCCD diffractometer	3355 independent reflections
Radiation source: fine-focus sealed tube	1633 reflections with *I* > 2σ(*I*)
Monochromator: graphite	*R*_int_ = 0.080
Detector resolution: 9 pixels mm^-1^	θ_max_ = 25.2º
*T* = 150(2) K	θ_min_ = 2.7º
φ scans and ω scans with κ offsets	*h* = −9→9
Absorption correction: multi-scan(SORTAV; Blessing, 1995)	*k* = −12→13
*T*_min_ = 0.768, *T*_max_ = 0.996	*l* = −14→14
7088 measured reflections	

### Refinement

**Table:**

Refinement on *F*^2^	Secondary atom site location: difference Fourier map
Least-squares matrix: full	Hydrogen site location: inferred from neighbouring sites
*R*[*F*^2^ > 2σ(*F*^2^)] = 0.063	H-atom parameters constrained
*wR*(*F*^2^) = 0.190	*w* = 1/[σ^2^(*F*_o_^2^) + (0.0923*P*)^2^] where *P* = (*F*_o_^2^ + 2*F*_c_^2^)/3
*S* = 0.96	(Δ/σ)_max_ < 0.001
3355 reflections	Δρ_max_ = 0.30 e Å^−^^3^
289 parameters	Δρ_min_ = −0.27 e Å^−^^3^
Primary atom site location: structure-invariant direct methods	Extinction correction: none

### Special details

**Table:**

Geometry. All e.s.d.'s (except the e.s.d. in the dihedral angle between two l.s. planes) are estimated using the full covariance matrix. The cell e.s.d.'s are taken into account individually in the estimation of e.s.d.'s in distances, angles and torsion angles; correlations between e.s.d.'s in cell parameters are only used when they are defined by crystal symmetry. An approximate (isotropic) treatment of cell e.s.d.'s is used for estimating e.s.d.'s involving l.s. planes.
Refinement. Refinement of *F*^2^ against ALL reflections. The weighted *R*-factor *wR* and goodness of fit *S* are based on *F*^2^, conventional *R*-factors *R* are based on *F*, with *F* set to zero for negative *F*^2^. The threshold expression of *F*^2^ > σ(*F*^2^) is used only for calculating *R*-factors(gt) *etc*. and is not relevant to the choice of reflections for refinement. *R*-factors based on *F*^2^ are statistically about twice as large as those based on *F*, and *R*- factors based on ALL data will be even larger.

### Fractional atomic coordinates and isotropic or equivalent isotropic displacement parameters (Å^2^)

**Table:**

	*x*	*y*	*z*	*U*_iso_*/*U*_eq_	
O1A	0.1429 (4)	0.0985 (3)	0.2676 (2)	0.0517 (8)	
O2A	0.4182 (4)	−0.0092 (3)	0.2921 (2)	0.0504 (8)	
O3A	0.3173 (4)	0.1684 (3)	0.1182 (2)	0.0524 (8)	
O4A	0.9007 (4)	0.1716 (3)	−0.2527 (3)	0.0685 (10)	
O5A	1.0631 (5)	0.1300 (3)	−0.0977 (3)	0.0690 (10)	
N1A	0.9198 (6)	0.1542 (3)	−0.1481 (4)	0.0498 (10)	
C1A	−0.1660 (6)	−0.0576 (4)	0.5216 (3)	0.0495 (12)	
H1A1	−0.1245	−0.1509	0.5502	0.059*	
H1A2	−0.2960	−0.0463	0.5211	0.059*	
C2A	−0.1003 (6)	0.0194 (4)	0.3939 (3)	0.0513 (12)	
H2A1	−0.1389	−0.0145	0.3396	0.062*	
H2A2	−0.1520	0.1112	0.3624	0.062*	
C3A	0.0979 (6)	0.0112 (4)	0.3927 (3)	0.0474 (12)	
H3A	0.1518	−0.0796	0.4145	0.057*	
C4A	0.3057 (6)	0.0763 (4)	0.2341 (4)	0.0453 (11)	
C5A	0.4739 (6)	0.1635 (4)	0.0559 (3)	0.0409 (11)	
C6A	0.6345 (6)	0.1517 (4)	0.1054 (4)	0.0476 (12)	
H6AA	0.6442	0.1459	0.1844	0.057*	
C7A	0.7824 (6)	0.1483 (4)	0.0383 (4)	0.0478 (11)	
H7AA	0.8962	0.1381	0.0709	0.057*	
C8A	0.7621 (6)	0.1599 (4)	−0.0766 (3)	0.0402 (10)	
C9A	0.6019 (6)	0.1746 (4)	−0.1267 (3)	0.0459 (11)	
H9AA	0.5926	0.1831	−0.2068	0.055*	
C10A	0.4527 (6)	0.1771 (4)	−0.0601 (3)	0.0455 (11)	
H10A	0.3391	0.1878	−0.0932	0.055*	
O1B	0.9940 (4)	0.5357 (3)	0.1688 (2)	0.0493 (8)	
O2B	0.9203 (4)	0.3799 (3)	0.3498 (2)	0.0568 (9)	
O3B	0.8673 (4)	0.5951 (3)	0.2963 (2)	0.0486 (8)	
O4B	0.4719 (5)	0.5191 (3)	0.7792 (3)	0.0708 (11)	
O5B	0.4665 (5)	0.7279 (4)	0.6849 (3)	0.0731 (11)	
N1B	0.5072 (5)	0.6198 (4)	0.6918 (3)	0.0527 (10)	
C1B	0.8165 (6)	0.4921 (4)	−0.0214 (4)	0.0507 (12)	
H1B1	0.7325	0.4509	−0.0465	0.061*	
H1B2	0.7480	0.5565	0.0008	0.061*	
C2B	0.9217 (6)	0.3884 (4)	0.0905 (3)	0.0500 (12)	
H2B1	0.8409	0.3535	0.1597	0.060*	
H2B2	0.9730	0.3162	0.0728	0.060*	
C3B	1.0669 (6)	0.4393 (4)	0.1269 (3)	0.0497 (12)	
H3B	1.1392	0.3659	0.1938	0.060*	
C4B	0.9251 (6)	0.4897 (4)	0.2793 (4)	0.0474 (11)	
C5B	0.7791 (6)	0.5888 (4)	0.4005 (3)	0.0425 (11)	
C6B	0.7732 (6)	0.4775 (4)	0.5097 (3)	0.0457 (11)	
H6BA	0.8299	0.3960	0.5189	0.055*	
C7B	0.6813 (6)	0.4894 (4)	0.6054 (4)	0.0473 (11)	
H7BA	0.6735	0.4149	0.6810	0.057*	
C8B	0.6022 (6)	0.6085 (4)	0.5905 (3)	0.0412 (10)	
C9B	0.6109 (6)	0.7178 (4)	0.4819 (3)	0.0439 (11)	
H9BA	0.5554	0.7996	0.4727	0.053*	
C10B	0.7007 (6)	0.7072 (4)	0.3872 (3)	0.0405 (10)	
H10B	0.7085	0.7822	0.3120	0.049*	

### Atomic displacement parameters (Å^2^)

**Table:**

	*U*^11^	*U*^22^	*U*^33^	*U*^12^	*U*^13^	*U*^23^
O1A	0.052 (2)	0.0532 (18)	0.0391 (16)	0.0002 (15)	0.0040 (14)	−0.0132 (14)
O2A	0.052 (2)	0.0445 (17)	0.0441 (16)	0.0074 (16)	−0.0042 (15)	−0.0133 (13)
O3A	0.055 (2)	0.0517 (17)	0.0347 (16)	0.0048 (15)	0.0035 (14)	−0.0093 (14)
O4A	0.061 (3)	0.087 (2)	0.072 (2)	−0.0174 (19)	0.0189 (18)	−0.048 (2)
O5A	0.045 (2)	0.064 (2)	0.078 (2)	−0.0100 (18)	0.0049 (19)	−0.0146 (17)
N1A	0.047 (3)	0.040 (2)	0.059 (3)	−0.0088 (19)	0.004 (2)	−0.0180 (18)
C1A	0.051 (3)	0.052 (3)	0.045 (2)	−0.015 (2)	0.003 (2)	−0.019 (2)
C2A	0.056 (3)	0.062 (3)	0.039 (2)	−0.008 (2)	−0.001 (2)	−0.025 (2)
C3A	0.055 (3)	0.051 (3)	0.036 (2)	−0.007 (2)	0.001 (2)	−0.019 (2)
C4A	0.049 (3)	0.046 (3)	0.044 (3)	−0.006 (2)	0.008 (2)	−0.025 (2)
C5A	0.043 (3)	0.035 (2)	0.041 (2)	−0.003 (2)	0.010 (2)	−0.0155 (18)
C6A	0.055 (3)	0.048 (3)	0.042 (2)	−0.003 (2)	−0.007 (2)	−0.022 (2)
C7A	0.043 (3)	0.048 (3)	0.052 (3)	−0.007 (2)	−0.005 (2)	−0.021 (2)
C8A	0.042 (3)	0.032 (2)	0.044 (2)	−0.0007 (19)	0.002 (2)	−0.0166 (18)
C9A	0.054 (3)	0.045 (2)	0.035 (2)	−0.010 (2)	−0.001 (2)	−0.0141 (19)
C10A	0.040 (3)	0.050 (3)	0.039 (2)	−0.008 (2)	0.000 (2)	−0.0126 (19)
O1B	0.059 (2)	0.0453 (16)	0.0435 (17)	−0.0044 (15)	0.0073 (14)	−0.0209 (13)
O2B	0.079 (3)	0.0435 (19)	0.0430 (17)	−0.0046 (16)	0.0087 (15)	−0.0169 (15)
O3B	0.063 (2)	0.0411 (17)	0.0404 (16)	−0.0016 (15)	0.0082 (14)	−0.0194 (13)
O4B	0.077 (3)	0.075 (2)	0.0455 (19)	−0.004 (2)	0.0170 (17)	−0.0174 (17)
O5B	0.084 (3)	0.072 (2)	0.079 (2)	−0.009 (2)	0.0228 (19)	−0.050 (2)
N1B	0.048 (3)	0.064 (3)	0.048 (2)	−0.003 (2)	0.0061 (18)	−0.029 (2)
C1B	0.051 (3)	0.049 (3)	0.054 (3)	−0.002 (2)	0.005 (2)	−0.027 (2)
C2B	0.066 (3)	0.045 (3)	0.042 (2)	−0.009 (2)	0.009 (2)	−0.022 (2)
C3B	0.066 (3)	0.042 (2)	0.042 (2)	−0.003 (2)	0.004 (2)	−0.021 (2)
C4B	0.060 (3)	0.043 (3)	0.036 (2)	−0.008 (2)	−0.001 (2)	−0.014 (2)
C5B	0.046 (3)	0.047 (3)	0.035 (2)	−0.007 (2)	−0.0012 (19)	−0.0187 (19)
C6B	0.048 (3)	0.039 (2)	0.051 (3)	0.002 (2)	0.000 (2)	−0.022 (2)
C7B	0.048 (3)	0.047 (3)	0.041 (2)	−0.008 (2)	0.001 (2)	−0.015 (2)
C8B	0.038 (3)	0.046 (3)	0.040 (2)	−0.005 (2)	0.0024 (19)	−0.020 (2)
C9B	0.043 (3)	0.043 (2)	0.050 (3)	−0.003 (2)	−0.003 (2)	−0.025 (2)
C10B	0.045 (3)	0.041 (2)	0.037 (2)	−0.010 (2)	0.0005 (19)	−0.0180 (19)

### Geometric parameters (Å, °)

**Table:**

O1A—C4A	1.327 (5)	O1B—C4B	1.328 (5)
O1A—C3A	1.469 (4)	O1B—C3B	1.472 (5)
O2A—C4A	1.199 (5)	O2B—C4B	1.184 (5)
O3A—C4A	1.352 (5)	O3B—C4B	1.352 (5)
O3A—C5A	1.404 (5)	O3B—C5B	1.398 (5)
O4A—N1A	1.223 (4)	O4B—N1B	1.236 (4)
O5A—N1A	1.224 (5)	O5B—N1B	1.222 (5)
N1A—C8A	1.475 (5)	N1B—C8B	1.463 (5)
C1A—C2A	1.518 (5)	C1B—C3B^ii^	1.507 (5)
C1A—C3A^i^	1.525 (6)	C1B—C2B	1.535 (5)
C1A—H1A1	0.9900	C1B—H1B1	0.9900
C1A—H1A2	0.9900	C1B—H1B2	0.9900
C2A—C3A	1.512 (6)	C2B—C3B	1.503 (6)
C2A—H2A1	0.9900	C2B—H2B1	0.9900
C2A—H2A2	0.9900	C2B—H2B2	0.9900
C3A—C1A^i^	1.525 (6)	C3B—C1B^ii^	1.507 (5)
C3A—H3A	1.0000	C3B—H3B	1.0000
C5A—C6A	1.365 (6)	C5B—C10B	1.370 (5)
C5A—C10A	1.379 (6)	C5B—C6B	1.388 (5)
C6A—C7A	1.379 (6)	C6B—C7B	1.395 (6)
C6A—H6AA	0.9500	C6B—H6BA	0.9500
C7A—C8A	1.373 (5)	C7B—C8B	1.373 (6)
C7A—H7AA	0.9500	C7B—H7BA	0.9500
C8A—C9A	1.358 (6)	C8B—C9B	1.375 (5)
C9A—C10A	1.381 (6)	C9B—C10B	1.372 (5)
C9A—H9AA	0.9500	C9B—H9BA	0.9500
C10A—H10A	0.9500	C10B—H10B	0.9500
			
C4A—O1A—C3A	116.0 (3)	C4B—O1B—C3B	116.4 (3)
C4A—O3A—C5A	118.0 (3)	C4B—O3B—C5B	123.4 (3)
O4A—N1A—O5A	123.8 (4)	O5B—N1B—O4B	123.8 (4)
O4A—N1A—C8A	118.7 (4)	O5B—N1B—C8B	118.3 (4)
O5A—N1A—C8A	117.5 (4)	O4B—N1B—C8B	117.8 (4)
C2A—C1A—C3A^i^	109.7 (3)	C3B^ii^—C1B—C2B	112.3 (4)
C2A—C1A—H1A1	109.7	C3B^ii^—C1B—H1B1	109.1
C3A^i^—C1A—H1A1	109.7	C2B—C1B—H1B1	109.1
C2A—C1A—H1A2	109.7	C3B^ii^—C1B—H1B2	109.1
C3A^i^—C1A—H1A2	109.7	C2B—C1B—H1B2	109.1
H1A1—C1A—H1A2	108.2	H1B1—C1B—H1B2	107.9
C3A—C2A—C1A	111.1 (3)	C3B—C2B—C1B	112.9 (4)
C3A—C2A—H2A1	109.4	C3B—C2B—H2B1	109.0
C1A—C2A—H2A1	109.4	C1B—C2B—H2B1	109.0
C3A—C2A—H2A2	109.4	C3B—C2B—H2B2	109.0
C1A—C2A—H2A2	109.4	C1B—C2B—H2B2	109.0
H2A1—C2A—H2A2	108.0	H2B1—C2B—H2B2	107.8
O1A—C3A—C2A	105.7 (3)	O1B—C3B—C2B	110.5 (4)
O1A—C3A—C1A^i^	108.5 (3)	O1B—C3B—C1B^ii^	106.1 (3)
C2A—C3A—C1A^i^	111.9 (4)	C2B—C3B—C1B^ii^	111.9 (3)
O1A—C3A—H3A	110.2	O1B—C3B—H3B	109.4
C2A—C3A—H3A	110.2	C2B—C3B—H3B	109.4
C1A^i^—C3A—H3A	110.2	C1B^ii^—C3B—H3B	109.4
O2A—C4A—O1A	127.8 (4)	O2B—C4B—O1B	127.6 (4)
O2A—C4A—O3A	126.7 (4)	O2B—C4B—O3B	127.0 (4)
O1A—C4A—O3A	105.5 (4)	O1B—C4B—O3B	105.3 (3)
C6A—C5A—C10A	122.8 (4)	C10B—C5B—C6B	121.5 (4)
C6A—C5A—O3A	122.0 (4)	C10B—C5B—O3B	113.0 (3)
C10A—C5A—O3A	115.1 (4)	C6B—C5B—O3B	125.5 (4)
C5A—C6A—C7A	118.6 (4)	C5B—C6B—C7B	117.8 (4)
C5A—C6A—H6AA	120.7	C5B—C6B—H6BA	121.1
C7A—C6A—H6AA	120.7	C7B—C6B—H6BA	121.1
C8A—C7A—C6A	118.8 (4)	C8B—C7B—C6B	120.1 (4)
C8A—C7A—H7AA	120.6	C8B—C7B—H7BA	120.0
C6A—C7A—H7AA	120.6	C6B—C7B—H7BA	120.0
C9A—C8A—C7A	122.5 (4)	C7B—C8B—C9B	121.2 (4)
C9A—C8A—N1A	118.4 (4)	C7B—C8B—N1B	119.6 (3)
C7A—C8A—N1A	119.0 (4)	C9B—C8B—N1B	119.2 (4)
C8A—C9A—C10A	119.3 (4)	C10B—C9B—C8B	119.2 (4)
C8A—C9A—H9AA	120.3	C10B—C9B—H9BA	120.4
C10A—C9A—H9AA	120.3	C8B—C9B—H9BA	120.4
C5A—C10A—C9A	117.9 (4)	C5B—C10B—C9B	120.2 (3)
C5A—C10A—H10A	121.0	C5B—C10B—H10B	119.9
C9A—C10A—H10A	121.0	C9B—C10B—H10B	119.9

Symmetry codes: (i) −*x*, −*y*, −*z*+1; (ii) −*x*+2, −*y*+1, −*z*.

### Hydrogen-bond geometry (Å, °)

**Table:**

*D*—H···*A*	*D*—H	H···*A*	*D*···*A*	*D*—H···*A*
C10A—H10A···O5A^iii^	0.95	2.32	3.205 (6)	155
C6B—H6BA···O2B	0.95	2.24	2.812 (5)	118
C9B—H9BA···O2A^iv^	0.95	2.48	3.184 (5)	131

Symmetry codes: (iii) *x*−1, *y*, *z*; (iv) *x*, *y*+1, *z*.
